# Student perceptions of reproductive health education in US medical schools: a qualitative analysis of students taking family planning electives

**DOI:** 10.3402/meo.v20.28973

**Published:** 2015-11-11

**Authors:** Kathryn Veazey, Claudia Nieuwoudt, Christina Gavito, Kristina Tocce

**Affiliations:** 1 Department of Emergency Medicine, Hennepin County Medical Center, Minneapolis, MN, USA; 2 School of Medicine, University of Colorado, Anschutz Medical Campus, Aurora, CO, USA; 3 Department of Obstetrics and Gynecology, University of Colorado School of Medicine, Aurora, CO, USA

**Keywords:** family planning, abortion, contraception, counseling, education

## Abstract

**Background:**

Abortion services will be sought by an estimated one in three US women before they reach age 45. Despite the importance of family planning (FP) care, many medical schools do not currently offer formal education in this area, and students are unable to meet associated competency standards prior to graduation.

**Purpose:**

The purpose of this study was to explore students’ motivations in pursuing FP electives throughout the United States, their experiences during these courses, and any impact of these rotations on their plans for future practice.

**Method:**

We conducted a qualitative study consisting of semi-structured interviews with medical students upon completing fourth-year FP electives at US medical schools. Thirty-seven LCME-accredited US medical schools offered fourth-year FP electives. Course directors at 21 of these institutions recruited study participants between June 2012 and June 2013. Interviews were transcribed, coded, and analyzed with ATLAS/ti software to identify salient themes.

**Results:**

We interviewed 29 students representing 14 institutions from all regions of the United States (East Coast, Midwest, South, and West Coast). Five central themes emerged. Medical students are using FP electives to fill gaps in the standard curriculum. Elective participation did not change students’ pre-elective stance on abortion. Many students intend to provide abortion in the future but identified possible limiting factors. Proficiency in contraception and options counseling were top competencies desired and gained. Students reported excellent satisfaction with FP electives and would recommend it to their peers, regardless of their personal beliefs.

**Conclusions:**

Interview data revealed that students are using FP electives to fill gaps within preclinical and clinical medical school curriculum. Future physicians will be unable to provide comprehensive care for their female patients if they are not provided with this education. Research should be directed at development and analysis of comprehensive FP curricula, which will allow students to obtain the knowledge necessary to best care for their patients.

Greater than half of all pregnancies in the United States each year are unintended, with 40% of these ending in abortion (1). An estimated 30% of US women will have an abortion by the time they reach age 45 (2), and the American College of Obstetricians and Gynecologists defines induced abortion as ‘an essential component of women's health care’ (3). Despite this, the number of abortion providers has been steadily declining. This underscores the necessity of training future providers and prioritizing making family planning (FP) education available in medical schools (4).

The Association of Professors of Gynecology and Obstetrics (APGO) provides clear objectives for medical students including: provide non-directive counseling to patients surrounding pregnancy options including unintended pregnancy, list surgical and non-surgical methods of pregnancy termination, identify potential complications of pregnancy termination, and describe the public health impact of the legal status of abortion (5). However, many medical schools do not provide the necessary training to meet these objectives. A 2005 study found that a third of US medical schools offered no formal abortion education (6). In 2010, we found that students at our own institution were largely unable to meet the APGO objectives by the end of their third year (7). Similar results were found in fourth-year medical students following an evaluation of student competence at a Canadian medical school (8).

Our study sought to examine students’ experiences with FP education through a nationwide qualitative investigation of fourth-year medical students following their completion of FP electives. We explored motivations to enroll in this elective, as well as the elective's influence on students’ beliefs and attitudes regarding abortion. We also examined students’ self-perceived competency with common FP topics, goals for future practice, and overall satisfaction post-elective. Based on the themes identified, we offer suggestions for addressing and navigating the barriers to FP education commonly faced in medical schools.

## Methods

US institutions offering FP electives were identified via internet search and through the National Office in Family Planning. For consistency, we required electives to be 2–4 weeks in duration to be eligible for inclusion. Course directors at the institutions identified were then emailed and invited to share our recruitment letter with their students during all FP elective orientations between April 1, 2012, and June 1, 2013. Reminder emails were sent to participating directors every 2 months.

The student recruitment letter asked students to contact us via email if they were interested in participating in a 45-min telephone interview about their motivations to enroll in the FP elective, perceptions of FP care during and following the elective, and their plans for practice. The letter also explained that participatory status would not be shared with course directors, data would be de-identified, and that participation would not influence their elective grade. A US$30 gift card was provided at the conclusion of the interview as compensation for time and commitment. The Colorado Multiple Institutional Review Board approved this research.

We identified 37 FP electives among the 141 Association of American Medical Colleges (AAMC)–accredited medical schools in the United States (9). Of these, six were excluded because they were not 2–4 weeks in duration. Ten course directors did not respond to the invitation. In total, 21 (68%) course directors agreed to share our recruitment letters with their students.

All interviews were conducted within 6 weeks of the end of a student's rotation. The interviewer identified herself as a medical student research assistant who had undergone training in qualitative research methods. Students were reminded that their grade would not be affected, nor their participation shared. The same individual conducted all of the semi-structured interviews using an interview guide consisting of 19 questions. The interviewer deviated from the questions whenever necessary to explore responses or to ask for clarification. Students were asked about what motivated them to pursue additional FP training, their goals for the rotation, how their experiences influenced their beliefs, attitudes and knowledge of FP, any challenges they faced during the rotation, their practice intentions, post-rotation competency with FP skills, and overall satisfaction with the elective. At the conclusion of the interview, demographic information including sex, age, relationship status, and race was collected.

All interviews were recorded and transcribed. The interviewer reviewed the transcriptions for accuracy. An investigator and a professional qualitative researcher independently coded each interview to determine common themes. The minimal incongruent coding was resolved by discussion between the coders. The data were analyzed for themes using ATLAS/ti qualitative analysis software, 7.1 (Scientific Software Development, Berlin, Germany). We use descriptive statistics to characterize the study sample and representative quotations to illustrate salient themes.

## Results

### Participant and course characteristics

Twenty-nine students from 14 institutions representing all regions of the United States (East Coast, Midwest, South, and West Coast) participated in the study. A 4-week course length was most common, with 19 students (66%) reporting taking their elective for 4 weeks. Twenty-seven students (93%) indicated that their elective was completed at an academically affiliated location, 11 students (38%) had experience at Planned Parenthood, and 14 (48%) spent time at a private, community, or free-standing clinic during the course. All students reported exposure to abortion counseling, contraception provision (including IUD insertion) and counseling, as well as administration of consent for these services.

Participants were primarily female (*n=*27, 93%), white (*n=*23, 79%), Christian (*n=*12, 41%), and single (*n=*12, 41%). Most students were interested in applying to a residency in obstetrics and gynecology (Ob/Gyn) (*n=*16, 55%) or family medicine (*n=*8, 28%). Two students were undecided between Ob/Gyn and another specialty, two planned a career in pediatrics, and one student intended to pursue psychiatry (Table 1).

**Table 1:** Demographic characteristics of the study sample

Characteristic (*N=*29)	Mean±SD or % (*n*)
Age (years)	27.6±3.1
Sex	
Female	93 (27)
Male	7 (2)
Race	
White	79 (23)
Black	10 (3)
Latino	3 (1)
Other	7 (2)
Religion	
Christian	41 (12)
No religion	21 (6)
Jewish	17 (5)
Atheist	7 (2)
Other	14 (4)
Relationship status	
Single	41 (12)
In a relationship	28 (8)
Married/engaged	28 (8)
Unclear	3 (1)
Intended specialty	
Obstetrics Gynecology	55 (16)
Family Medicine	28 (8)
Other (Psychiatry, Pediatrics)	10 (3)
Undecided	7 (2)

Five central themes were identified from the interviews, including:

- Motivations for participating in and specific goals for the elective.
- Beliefs about and attitudes toward abortion before and after completing the elective.
- Intention to provide abortion care in future practices and concerns associated with provision of this care.
- Changes in self-perceived competency following the elective.
- Influence of overall satisfaction and likelihood of recommending the elective to peers.

### Motivations for choosing and goals for the elective

When asked about their motivations for choosing an FP elective, 21 students (72%) described a need for greater exposure to FP care. Fourteen students (48%) indicated that this elective was necessary to obtain knowledge not available during their third-year clerkships and sought to use this knowledge to inform their residency program choices. For example, a fourth-year student described:

… I wanted to learn more about it before I started residency so I would know how important it was to make it a priority in my residency training … I also just wanted exposure because I didn't get much in my regular OB rotation.

Six students (21%) mentioned that a desire to provide abortion in their future practice was a primary motivating factor. Other areas of motivation included exposure to terminations and how to do them (*n=*6, 21%), future specialty choice in Ob/Gyn or Family Medicine (*n=*10, 34%), and a special interest in women's health (*n=*2, 7%), adolescent reproductive health (*n=*3, 10%), and/or teen contraception (*n=*3, 10%). Values clarification was also cited by seven students (24%). A student described using the rotation to define personal beliefs surrounding abortion practice:

I would say part of my reason for taking the elective was to … see how I felt actually doing as opposed to talking about it [abortion] academically or saying that women should have the right to make choices about their own body.

The most common goals for the elective were to increase knowledge of abortion (*n=*21, 72%), contraceptive technology and counseling (*n=*13, 45%), as well as experience with abortion care (*n=*15, 52%). Students chose the elective to acquire skills in FP and to increase their comfort level with provision of care (*n=*6, 21%). Other specific skill-based goals included IUD insertion (*n=*5, 17%) and pregnancy termination (*n=*6, 21%). For example:

I sort of knew that it would be … more based on procedural … with regards to abortions … but really my, at least my hope for the elective is that I would really get pretty viable experience in doing counseling; like options counseling, contraceptive counseling, kinda learning the language to talk about what can be a kind of sensitive topic. So I figured I would be at least somewhat involved with the procedural side of things … I was hoping that I would be very involved with the counseling side.

### Influence on personal beliefs and attitudes

The majority of students (*n=*27, 93%) indicated that the elective did not substantially change their personal beliefs and attitudes toward FP care; however, many students felt that their pro-choice views were strengthened. Students used a variety of terms to describe the impact of the course, including: ‘reconfirmed’, ‘reinforced’, ‘solidified’, ‘challenged’, ‘tested’, ‘more defined’, ‘firm’, ‘normalized’, and ‘expanded upon’. Students also discussed greater understanding of the need for increased access to abortions and the importance of being able to counsel women regarding their options (*n=*18, 62%). One student felt less judgmental about women who choose to have abortions.

It [FP elective] confirmed my stance on being pro-choice and fighting for women's rights. It kind of motivated me to be more open about pro-choice and abortion, especially among classmates and future colleagues because it's while I felt like it was taboo, a taboo topic growing up, I would like to be more open about it now moving forward in my career.

Although students’ attitudes were not altered in favor of less access to FP and abortion, one student stated that his or her beliefs had become less clear with regard to the legality of abortion, and gestational age limits in particular. The student said:

I was a little bit more uncertain about my opinion about later term procedures and if there should be a like line where the line should be drawn about the legality and when they should be allowed and I don't know that I have a very clear answer now….

### Intentions to provide abortion in future practice

Most students (*n=*25, 86%) indicated an intention to provide abortion care in their future practices. Students noted that this would be contingent on receipt of adequate training as residents and sufficient patient volume to ensure competency. They acknowledged that quality training would be crucial to patient safety, and several students indicated that this influenced their choice of residency program. One student stated:

I think it just reaffirms my desire to end up at a residency program that I will learn comprehensive family planning and makes me seek out a residency program that will fit my values.

When asked about potential deterring factors, students mentioned a variety of social and community issues. These reasons included community climate, provider stigma, and legal and personal safety concerns. Overall, students felt that residency training and post-residency employment would determine their ability to provide abortion care.

Of the three students who stated that they did not intend to provide abortion, two indicated this was due to their specialty choice (pediatrics). The only student who did not plan to provide abortions for personal reasons cited religious beliefs that prevented him/her from providing abortion. However, this student was pro-choice.

### Self-perceived post-elective competency

More than half of students (*n=*15, 52%) worked from a written or verbal list of course objectives rather than specific competencies. A few students (*n=*8, 28%) received an outline of competencies for the elective and only five students (17%) mentioned any formal testing or other standardized assessment of their skills. When asked to evaluate the strengths gained from the course, the majority of students (*n=*22, 76%) reported greater competency in contraceptive counseling. Nearly half (*n=*14, 48%) reported increased competency in options counseling. A third of students (*n=*10, 34%) noted greater strengths in their general FP knowledge and seven students (24%) noted greater strengths in their termination-specific knowledge. A minority of students reported skills specific to termination procedures as skills gained (*n=*6, 21%). These skills cited as new strengths included LARC insertion (*n=*9, 31%) and termination procedures (*n=*6, 21%) (Table 2).

**Table 2:** Subjective competencies gained

Competency gained	% (*n*)^a^
Contraceptive counseling	76 (22)
Pregnancy options counseling	48 (14)
General FP knowledge	34 (10)
LARC insertion	31 (9)
Elective termination-specific knowledge	24 (7)
Performing abortions	21 (6)

a Percentages do not add to 100% as students could list >1 competency gained.

### Overall satisfaction

Every student interviewed (*n=*29, 100%) said that he or she would recommend the elective experience to his or her peers. Students indicated the FP elective offered excellent opportunities for active experience with procedures, large patient volume, and exposure to a wide variety of topics in FP. Students appreciated the hands-on nature of the elective, and felt their decision-making processes and autonomy were valued. Other reasons students would recommend the elective included exposure to contraceptive and options counseling, high standard of faculty mentors, and the opportunity to hear a variety of patients’ perspectives.

I think it's a really comprehensive experience, I think it exposes you to aspects of medicine that we don't learn in medical school that are really important.

In terms of personal beliefs, only three students (10%) said that they would not recommend the elective to anti-choice/pro-life students or at least felt that these students needed to approach the elective with an open attitude and see it as an opportunity to challenge their beliefs. Four students (14%) specifically stated that being pro-choice was not necessary to take the elective. Students felt that peers interested in Ob/Gyn, family medicine, internal medicine, and pediatrics would benefit from the elective; however, some students felt that this course would be beneficial to all of their peers regardless of specialty of interest.

## Discussion

It's a shame that we don't learn these skills or aren't required to learn these—or at least talk about these skills in any of the rotations.

This study was designed to elucidate the motivations and experiences of medical students enrolling in FP electives. After completing their standardized medical school curriculum, students should feel proficient in the basic skills of all core specialties. These skills include contraception and pregnancy options counseling and are incorporated into the APGO learning objectives for all medical students. Our interviews revealed a self-perceived lack of competency among FP elective students; the most commonly cited motivation for enrolling in an FP elective was a desire to gain needed exposure to FP topics that was not achieved through the standard preclinical or clinical curriculum. Rather than using the FP course to enrich baseline knowledge and experience, these students utilized this elective to fill gaps in their training.

The common themes that emerged from our interviews are consistent with existing literature. The FP curricular gap is supported by a national study that found 44% of AAMC member schools did not offer any formal education in FP during the preclinical years and 23% failed to offer abortion education during the third-year Ob/Gyn clerkship (6). A survey of Medical Students for Choice student coordinators found the inclusion of FP topics in the preclinical years to be extremely variable, with 33% of schools failing to include abortion in the preclinical curriculum. Less than 30% of all US medical schools represented included education regarding abortion options counseling, availability, policy, and post-procedure care in the pre-clinical years (10). This lack of education exists despite data demonstrating students’ interest in gaining knowledge related to FP and abortion (11, 12). Furthermore, our research reveals that only 26% of US medical schools offer an FP elective, indicating limited opportunity for students interested in FP, regardless of motivating factors.

Although these electives may remedy these educational disparities (13, 14), this was not their intended purpose. All medical students should strive to meet the APGO objectives: provide non-directive counseling to patients surrounding pregnancy options including unintended pregnancy, list surgical and non-surgical methods of pregnancy termination, identify potential complications of pregnancy termination, and describe the public health impact of the legal status of abortion (5). If students need to seek an elective to meet these objectives, they will either do so at the expense of other opportunities, or remain at a disadvantage. As our prior research demonstrated, third-year students with interests in a variety of specialties were not able to meet these objectives (7). Although one institution's findings cannot be extrapolated to all programs, they should prompt other institutions to evaluate their curricula for similar deficiencies. Standardization of FP curricula will ensure that all medical students receive adequate exposure to FP care, and meet the APGO objectives.

If students are unable to meet the FP learning objectives outlined by APGO, and standard clinical experiences for all students are not possible, other modalities should be utilized. The optimal combination of didactics, workshops, small group sessions, and simulation has yet to be defined; however, each of these modalities may offer significant FP curricular enhancement. Future research, including program evaluation, will help guide programs in implementing the most effective changes.

Physicians in all specialties need basic FP knowledge to provide comprehensive care and counseling to their patients, as women of reproductive age make up a significant portion of the population (62 million as of the most recent National Survey of Family Growth) (15). Research indicates that women value knowledgeable providers who respect their autonomy, and share unbiased information (16). Nearly all (96%) of women who received pregnancy options counseling found it helpful (17). All physicians are likely to encounter a patient requiring FP care (or needing referral for such care) at some point in their careers. Without adequate education, they will not be able to accomplish this, and patient care will be inadequate.

Although our study provides an in-depth view of student experiences in various geographic areas throughout the United States, it is limited by several factors. Our sample is self-selected, including only students who chose to take an elective in FP. The perceived competency in the general population of medical students is unknown. The overall response rate from FP elective course directors was relatively low (68%). It is possible that students in the non-responding programs would have expressed different viewpoints than those in our study, particularly as we do not have specific details regarding the FP curriculum at these institutions. We also do not know if the students who responded to our recruitment letter are different than those who did not. Thus our findings are not intrinsically generalizable.

Despite these limitations, our findings indicate that students with an active interest in women's health are not able to meet basic FP objectives after completing their standard clinical curricula. All medical students are expected to meet these objectives, regardless of their specialty choice. Future physicians will be unable to provide comprehensive care for their female patients if they are not provided with this education in medical school. Additional research should be directed at development and analysis of comprehensive FP curricula, which will allow all students to obtain the knowledge necessary to best care for their patients.
